# Supplementary material for: Geometric control of diffusing elements on InAs semiconductor surfaces via metal contacts
Source: Nat Commun. 2023 Jul 27;14:4541. doi: 10.1038/s41467-023-40157-5 (PMC10374539; doi:10.1038/s41467-023-40157-5)
Supplement: Supplementary file 1 — Supplementary Information [file 41467_2023_40157_MOESM1_ESM.pdf]

Supplementary Information

Geometric control of diffusing elements on InAs semiconductor surfaces via metal contacts

Benter et al.

Supplementary Note 1

Annealing the InAs(111)B substrate above 650°C inhibits the formation of a droplet-free zone around the Al/Pd metal stacks (see Supplementary Fig. 1). Due to the larger amount of excess In being released from the sample surface in a short time, the sink effect of the lithography pattern vanishes. The Pd layer is completely saturated with free In atoms. However, a shadow effect close to the metal is visible (white arrows in Supplementary Fig. 1a). This is a result of the metal stack on the surface and the main movement direction of the In droplets induced by the underlying crystal structure (here bottom right corner to upper left corner). Interestingly, the In droplets encountering the metal edge on one side are blocked from passing through this region. It becomes obvious that no In atom is released once it encounters Pd atoms. Droplets on the other side of the metal are solely formed by freed In on the InAs substrate close to pattern consequently moving away from the metal edge.

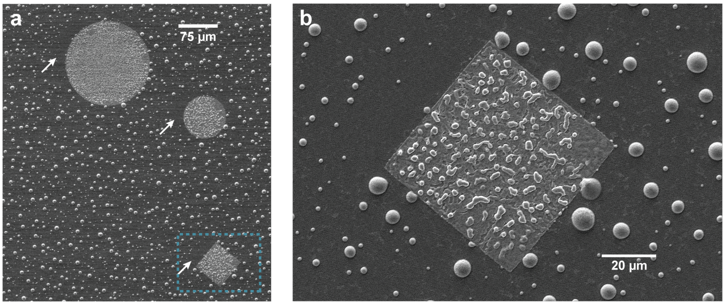

Supplementary Figure 1 Temperature threshold SEM images of a sample being annealed to 650°C. White arrows in a) indicating the shadowing effect induced by the presence of the metal pattern. b) is a zoom-in of the outlined region in a).

Supplementary Note 2

As indicated in Supplementary Fig. 2, for different shapes of the metal pattern employed in this study the droplet-free zone can be estimated with an ellipse. The aspect ratio between length and width of the square pattern is similar to the aspect ratio of a circle. Sharp features like the corners are not reproducible for the outline of the droplet-free zone (DFZ). We expect the appearance of the DFZ to change, and therefore a change in the aspect ratio of both axes of the ellipse, with a drastic change of the aspect ratio of the metal pattern.

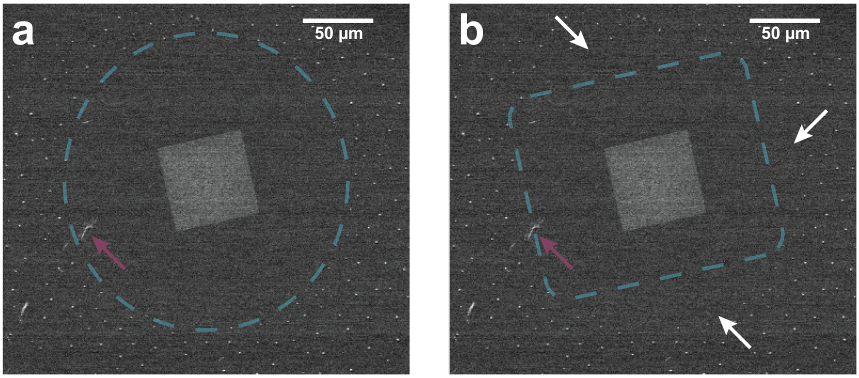

Supplementary Figure 2 Shape of droplet-free zone SEM images with different fitting of the droplet-free zone: (a) elliptical and (b) rectangular. White arrows indicate the missing DFZ for the rectangular fit in b. Red arrows indicated presence of nanostructures deposited on the substrate, which are not relevant to the outline of the DFZ.

Whether the boundary of the DFZ is closer to a circle or an ellipse depends on the aspect ratio of the lithography pattern, which can be seen in Supplementary Fig. 3. Increasing the aspect ratio results in a more elliptical appearance of the DFZ edge. The shape is also influenced by the diffusion coefficient of the free In atoms on the InAs(111)B substrate. For smaller diffusion coefficients the DFZ will decrease significantly tracing the original metal shape with greater detail.

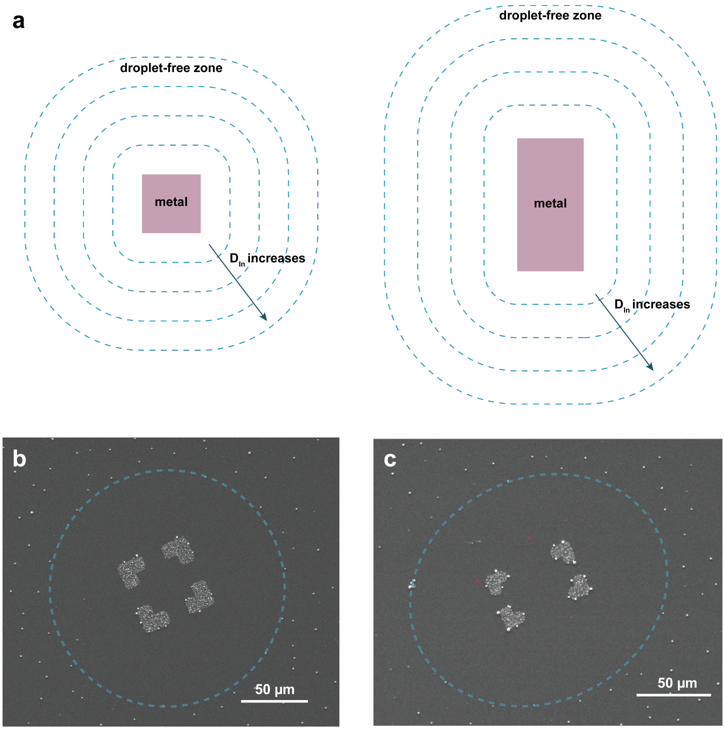

Supplementary Figure 3 Size of droplet-free zone a) Sketch of the development of the DFZ for different diffusion coefficients (D_{In} and metal pattern shapes.b) and c) are SEM images of two square patterns with different ratio aspects demonstrating the influence of shape of the lithography pattern on the DFZ zone.

Supplementary Note 3

Supplementary Fig. 4 showcases the difficulty to acquire an X-ray Photoemission Electron Microscopy (XPEEM) dataset for the *Pd 3d* core level. Due to the very low cross-section for photon energies below 150 eV focusing becomes problematic. As a result, small individual features within the metal stack displaying different metal compositions can not be resolved.

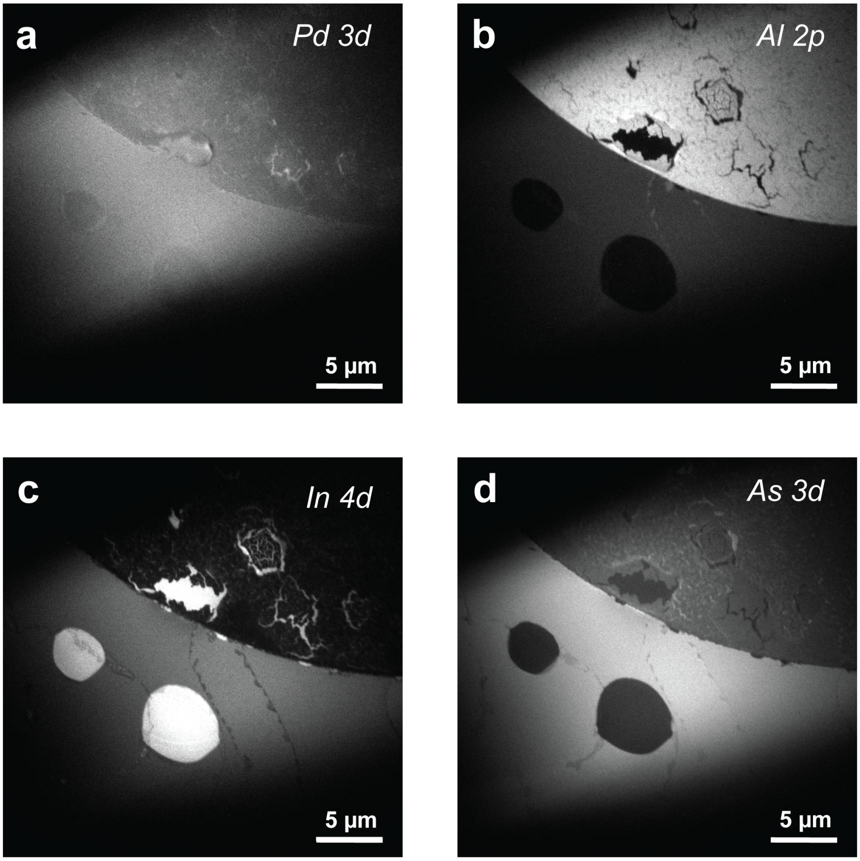

Supplementary Figure 4 Overall chemical composition at the metal edge XPEEM images for individual core levels at specific binding energies (a - 344 eV, b - 79.4 eV, c - 22 eV and d - 45.6 eV) chosen to display the highest contrast between areas with different chemical environments. Visible area is the same as in Fig. 2e (in the main text) and 6.

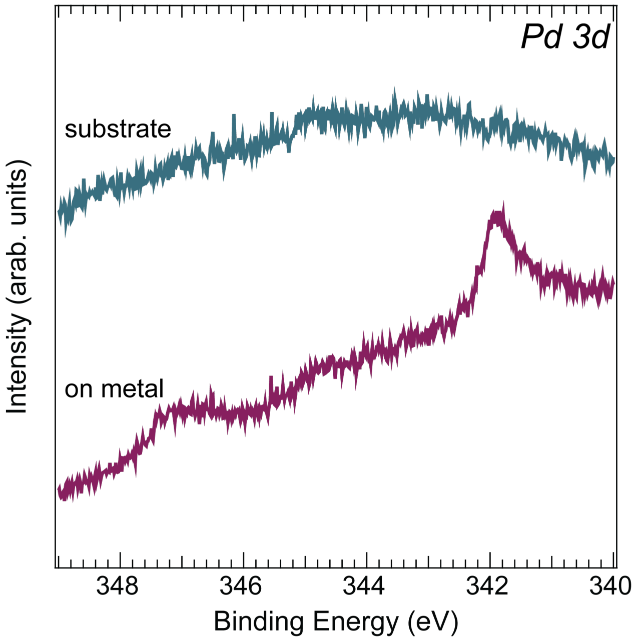

Supplementary Figure 5 Presence of Pd on the sample Raw data of µ-XPS measurements on the InAs(111)B substrate (top) and the Al/Pd metal stack (bottom) after annealing indicating clearly that the metal is confined to the original lithography pattern. Source data are provided as a Source Data file.

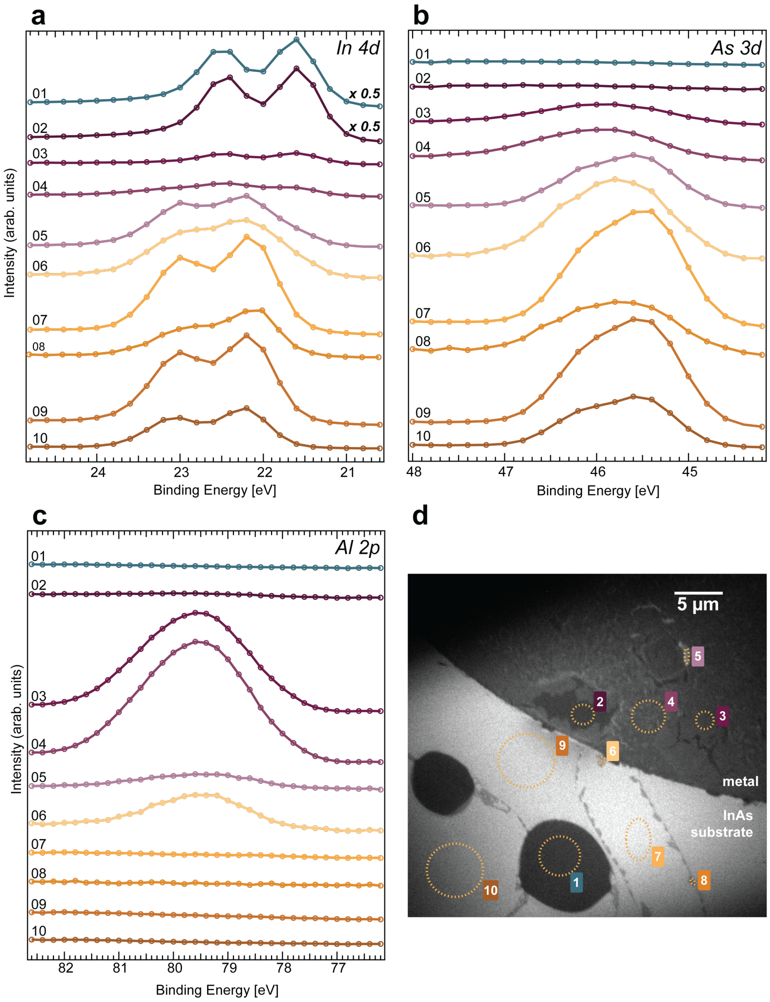

Supplementary Figure 6 XPEEM spectra for detailed chemical composition at the metal edge *In 4d* (a), *As 3d* (b) and *Al 2p* (c) core level extracted from measurement positions indicated in (d) via XPEEM image analysis. The spectra are shown with their original intensities except for 01 and 02 in (a), which are multiplied by 0.5. The metal stack consists of 5 nm Al and 20 nm Pd. Source data are provided as a Source Data file.

Supplementary Note 4

To determining the In concentration within the field of view, spectra from XPEEM image sequences were analysed for the *In 3d* core level. For all spectra a Shirley background was removed and the area below the peaks integrated. Position 1, 2, 7 and 9 in Supplementary Fig. 6d were taken as reference points, since the areas with metallic In (dropelt-1 and within the metal-2) correspond to a concentration of 100% In, and the InAs substrate (7 and 9) consists of 50% As and In atoms, respectively. Supplementary Fig. 7 displays a linear fit forced through the origin. The yellow rectangle marks the intensity regime determined for area 4 and 3 in Supplementary Fig. 6d. Therefore, we estimate an In concentration within the Pd layer of about 10% for the largest domain in the field of view.

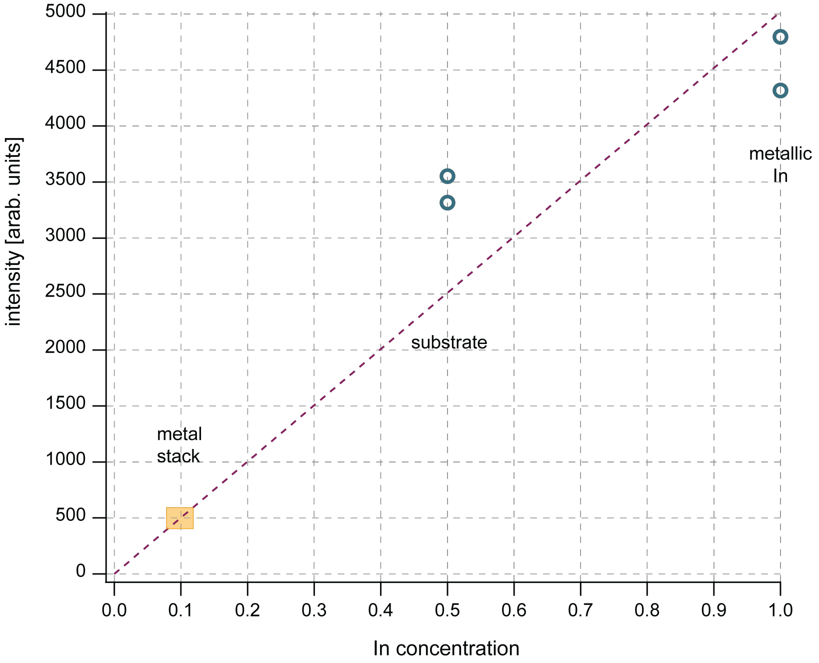

Supplementary Figure 7 Indium concentration over the sample Approximation of Indium concentration within the main domain of the metal stack visible in Supplementary Fig. 6. Source data are provided as a Source Data file.

Supplementary Note 5

Here, we propose a method to explain the droplet-free zone and to estimate its radius r_i. We model the concentration of In atoms and the concentration of stable, observable In droplets at the surface. As these form via a nucleation process, we use a reduced rate equation [1] approach, where In atoms can diffuse into the Pd layer, if they are close enough, or contribute to the nucleation of stable In droplets. The equation for the concentration of In atoms n_1 is given by

$$\frac{\partial n_1}{\partial t} = D\nabla^2 n_1 + F - \frac{n_1}{\tau} \tag{1}$$

where D is the diffusivity of In atoms along the surface, F the surface decomposition rate and τ the mean residence time of an In atom at the surface. For a circular Pd pattern with radius r_m, equation 1 should be solved for $r \geq r_m$ with $\nabla^2 = \partial^2/\partial r^2 + r^{-1}\partial/\partial r$. For sufficiently short times, before the Pd layer is saturated with In and before stable In droplets have started to coalesce, we can consider the steady state solution to eq. 1 with the boundary condition $n_1(r_m) = 0$,

$$n_1(r) = F\tau\left(1 - \frac{K_0(r/\lambda)}{K_0(r_m/\lambda)}\right) \tag{2}$$

where $K_0(x)$ is the modified Bessel function of the second kind and λ the In atom surface migration length, given by $\lambda = \sqrt{D\tau}$.

In the reduced rate equation approach, a steady state is assumed for all subcritical droplets and the density of critical droplets are given by a truncated Walton relation [1, 2],

$$\frac{n_i}{n_s} = \left(\frac{n_1}{n_s}\right)^i \exp\frac{E_i}{kT} \tag{3}$$

where n_s is the density of sites, i the size of a critical droplet meaning a droplet with equal probabilities for growth and decay. The binding energy for a critical droplet E_i is size dependent, for sufficiently large droplets ($i > 8$) modeled as $E_i = E_c(i - 2i^{2/3})$. In addition, k is the Boltzmann's constant and T the absolute temperature.

The densities of stable droplets are all reduced into one density $n_c = \sum_{j>i}^{\infty} n_j$, and their formation rate is calculated as

$$\frac{dn_c}{dt} = \sigma D n_1 n_i \tag{4}$$

where σ is a capture number [1], which is a slowly varying function of the droplet size (in the range of 5-10). Integration of eq. 4 and combination with eq. 3 results in

$$n_c = \sigma D t n_s^{1-i} n_1^{1+i} \exp\frac{E_i}{kT} \tag{5}$$

Note, n_c is distance dependent through n_1 (see eq. 2) and time dependent due to the integration.There are a number of more or less free parameters (i, E_i, σ, D and λ), which make the fitting process ambiguous. This in turn prevents the extraction of exact results. However, we can draw the conclusion that the In droplets form via a nucleation process and the critical size is large. In order to fit our experimental observation, we have set $E_c = 0.1\, eV$ and $\sigma = 10$. Next, we choose D and λ so that $n_1 < n_s$ with $n_s = 6.3 \times 10^{14}\ cm^{-2}$ on an InAs(111)B surface. Following this, we find a value of i, so that the value of n_c far away from the Pd layer agrees with the experimentally observed droplet density ($2.4 \times 10^5\ cm^2$ for $T = 550°C$, $t = 20\ min$, $r_m = 40\ \mu m$ and $r_i = 120 \mu m$), possibly after a small adjustment of λ. In addition, n_c should be essentially zero for r smaller than the experimentally observed r_i. If not, D and λ have to be adjusted and a new value for i has to be calculated. The curves in Supplementary Fig. 3 in the main text are calculated with $D = 8.7 \times 10^{-7}\, cm^2 s^{-1}$, $\lambda = 60.1\ \mu m$ and $i = 39$.

Supplementary Note 6

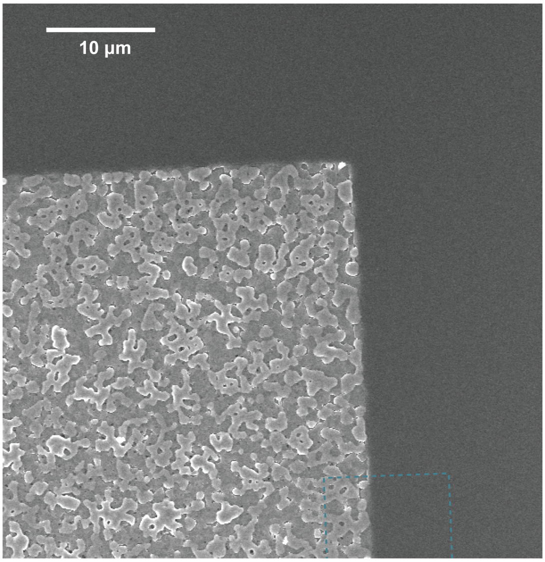

Supplementary Figure 8 Corresponding SEM image to AFM study SEM image of an metal stack edge (5 nm Al topped by 20 nm Pd) after annealing rapidly to 600°C. The encircled area corresponds to the area measured by AFM in Fig. 2c in the main text.

Supplementary Note 7

So far, we investigated the impact of rapid annealing processes on the formation and distribution of In droplets on InAs(111)B substrates in the vicinity of Al/Pd metal stacks. Here, the sample temperature was raised to a maximum of 600°C in about 1.5 min. The obtained results demonstrate the suitability of our approach to inhibit the presence of In droplets close to the metal stack for fast process steps involving high-temperatures (e.g. flash lamp annealing) for device manufacturing.

Considering high-temperature processes with longer time span, the temperature range to prevent In droplets should be chosen more carefully. For an *in-situ* study, a sample was kept at about 550°C for 24.5 min. In comparison to the rapid annealing process (about 4 min for ramping the temperature up and down, reference temperature 350°C) utilized before, we observe a distinct difference. Here, the DFZ vanishes over time and the substrate reaches a similar state like heating it rapidly to 650°C (compare images in section 1 with the movie in the SI). This is in agreement with our model as we should reach a state when the metal pattern is saturated with atoms coming from the InAs(111)B substrate. Furthermore, droplets, several µm in diameter, as well as their trails are present all over the surface. Interestingly, a shadowing effect close to the deposited metal patterns evolves resulting in a small area on one side where no droplets are present (see Fig. 1 in the main text). This is a result of two key features: (i) the dominant movement direction of the droplet due to the crystal structure of the sample, and (ii) In atoms alloyed with the Pd are not being released from the metal stack. This prevents a continuous flow of In atoms. Therefore, droplets need to be newly formed on the 'backside' of the metal pattern solely from excess In atoms freed from the substrate and not from the metal alloy. These will follow the main movement direction leading to an area at the metal edge deprived of droplets.

Supplementary Video

In-situ measurement were performed at a sample temperature of 550°C after removing the native oxide. The field of view is focused on the edge of an Al/Pd stack (5nm/20nm). The whole movie is recorded over 24.5 min in mirror mode. As observed before, In droplets are not stationary but move along a distinct crystal direction while accumulating additional atoms from the surface [3–5]. However, a minimum droplet size is necessary in order to shift them forward. Our *in-situ* experiments show the thermal etching of the individual terraces visible in the movie due to the high resolution (step edges move towards the left upper corner). This process frees up a large amount of As and In atoms which go into gas phase and stay on the surface, respectively. Nonetheless, only nm-sized droplets form in the vicinity of the metal edge. This indicates that not enough excess In atoms are available. This deprivation can only be explained by the presence of the Al/Pd stack. Therefore, a steady flow of individual In atoms into the metal pattern has to take place. Distinct changes in the appearance

of different domains in the metal during the annealing process indicate that substrate atoms are alloying into the stack (as discussed above). This clearly prevents the formation of µm-sized droplets in the DFZ for short annealing times. Longer process times will eventually enable movement of droplets into the DFZ, as well as localized droplet formation close to the metal. However, even though the appearance of the droplet-free zone can be counteracted by this, two additional effects become apparent. First, moving droplets that do not exceed a critical size will start to decrease significantly when close to the metal pattern even at sample temperatures that promote droplet formation and movement further away. Secondly, when lowering the sample temperature to slow down the mobility of the In droplets, droplets close to the metal will start to shrink independent of their original size until no significant amount of In is left at this position. Both effects illustrate that the main influence on the formation, size and movement of In droplets in the DFZ is the Al/Pd metal pattern. Therefore, we can conclude that the presence of this metal stack is inhibiting the existence of metallic In droplets on the InAs(111)B substrate in it's vicinity for temperatures below 650°C. Here, dependencies on sample temperature and annealing time influence the size of the DFZ.

Supplementary References

[1] Venables, J.A., Spiller, G.D.T., Hanbucken, M.: Nucleation and growth of thin films. Reports Prog. Phys. **47**(4), 399–459 (1984). https://doi.org/10.1088/0034-4885/47/4/002

[2] Walton, D.: Nucleation of vapor deposits. J. Chem. Phys. **37**(10), 2182–2188 (1962). https://doi.org/10.1063/1.1732985

[3] Kanjanachuchai, S., Photongkam, P.: Dislocation-Guided Self-Running Droplets. Cryst. Growth Des. **15**(1), 14–19 (2015). https://doi.org/10.1021/cg5013704

[4] Tersoff, J., Jesson, D.E., Tang, W.X.: Running Droplets of Gallium from Evaporation of Gallium Arsenide. Science **324**(236) (2009). https://doi.org/10.1126/science.1169546

[5] Hilner, E., Zakharov, A.A., Schulte, K., Kratzer, P., Andersen, J.N., Lundgren, E., Mikkelsen, A.: Ordering of the Nanoscale Step Morphology As a Mechanism for Droplet Self-Propulsion. Nano Lett. **9**(7), 2710–2714 (2009)
