# Supplementary material for: Geometric control of diffusing elements on InAs semiconductor surfaces via metal contacts
Source: Nat Commun. 2023 Jul 27;14:4541. doi: 10.1038/s41467-023-40157-5 (PMC10374539; doi:10.1038/s41467-023-40157-5)
Supplement: Supplementary file 3 — Description of Additional Supplementary Files [file 41467_2023_40157_MOESM3_ESM.pdf]

## Description of Additional Supplementary Files

Supplementary Movie 1
Description: *In-situ* measurement were performed at a sample temperature of 550°C after removing the native oxide. The field of view is focused on the edge of an Al/Pd stack (5nm/20nm). The whole movie is recorded over 24.5 min in mirror mode.
